# Cyclin A2 regulates homologous recombination DNA repair and sensitivity to DNA damaging agents and poly(ADP-ribose) polymerase (PARP) inhibitors in human breast cancer cells

**DOI:** 10.18632/oncotarget.20412

**Published:** 2017-08-24

**Authors:** Wei Wei Gu, Jie Lin, Xing Yu Hong

**Affiliations:** ^1^ Department of Vascular Surgery, China-Japan Union Hospital of Jilin University, Changchun, China; ^2^ Department of Hepatopancreatobility Surgery, China-Japan Union Hospital of Jilin University, Changchun, China

**Keywords:** cyclin A2, homologous recombination repair, double-strand breaks, MRE11, RAD51

## Abstract

Defects in homologous recombination (HR) repair are found in breast cancers. Intriguingly, breast cancers with defective HR show increased sensitivity to DNA crosslinking agents and poly(ADP-ribose) polymerase (PARP) inhibitors. As such, genes that can affect HR functions have been of high interest in studies aiming to develop biomarkers for predicting response to treatment with these agents. Cyclin A2 is a key component of the core cell cycle machinery. However, whether cyclin A2 dysfunctions could cause HR defect and mediate sensitivity to DNA damaging agents remain unclear. Here we show that loss of cyclin A2 causes high rates of double-strand breaks (DSB) in MCF-7 and MDA-MB-231 cells. The increased DSB was due to defective HR-mediated repair of the breaks, resulting from reduced MRE11 and RAD51 proteins. Cyclin A2 mediates MRE11 abundance through its MRE11 mRNA binding property and RAD51 abundance through inhibition of proteasome degradation of RAD51. Moreover, cyclin A2 depletion hypersensitized the cells to DNA damaging agents, such as cisplatin and melphalan. Our results demonstrate novel roles for cyclin A2 in regulating HR repair and determining sensitivity to DNA cross linkers and PARP inhibitors in breast cancer cells.

## INTRODUCTION

Cyclin A2, the ubiquitously expressed mammalian A-type cyclin, is a key component of the core cell cycle machinery. Cyclin A2 is known to play important roles in DNA replication and mitosis [1, 2]. In S-phase, cyclin A2 associates with CDK2 and regulates the initiation and progression of DNA replication [3, 4]. In the G2/M phase, it associates with CDK1 and controls both the nuclear and centrosomal mitotic events, favoring the entry into, and error-free progression through, mitosis until pro-metaphase [4-7]. Cyclin A2 is essential in embryonic and hematopoietic stem cells, but is dispensable for proliferation of fibroblasts because of the compensatory roles of other cyclins that are present in the cell cycle phases where cyclin A2 exists [8]. Whether there are any specific roles for cyclin A2 in DNA replication and mitosis that could potentially be targeted for cancer treatment remain unclear.

A recent study by Kanakkanthara et al. (2016) showed that cyclin A2 has a CDK-independent RNA binding property that is critical for the maintenance of efficient DNA repair in cells [9]. Cyclin A2 specifically binds to the MRE11 mRNA and promotes its translation to maintain sufficient levels of MRE11 and RAD50, two components of the MRN complex that is crucial for DSB repair [9, 10]. As such, low levels of cyclin A2 perturbed MRE11 translation and caused decreased abundance of both MRE11 and RAD50 proteins, leading to deficient DNA repair and high occurrence of DNA double-strand breaks [9].

In mammalian cells, the homologous recombination (HR) repair pathway facilitates highly accurate DSB repair [11]. The DNA end resection at DSBs sites is one of the initial steps of HR repair [12]. The MRN complex, involving MRE11-RAD50-NBS1 proteins, that binds to one or both sides of the DSBs has a central role in DNA end resection [10, 12-14]. After binding, RAD50’s coiled-coil arms stabilize the break, which is followed by MRE11 dimer-mediated close range stabilization, where required nuclease activities to initiate DNA end resection are provided by MRE11 [10, 15].

HR deficiency is of high therapeutic relevance in breast cancer as both platinum-based chemotherapies and Poly (ADP-ribose) polymerase (PARP) inhibitors have been found to be effective in HR-defective tumors [16, 17]. As such, there has been much interest in studies aiming to identify genes that affect HR pathway. Although cyclin A2 expression was found to be altered in human tumors [18-28], whether its deficiency would cause HR defect and sensitize cells to chemotherapeutics, remains unknown.

The aim of the present study, therefore, was to investigate whether cyclin A2 deficiency perturbs HR pathway in human breast cancer cells and to determine if its loss hypersensitizes the cells to DNA crosslinking agent, cisplatin, and PARP inhibitors, veliparib and olaparib.

## RESULTS

### Cyclin A2 siRNAs specifically suppress cyclin A2 mRNA and protein abundance

**Figure 1:**
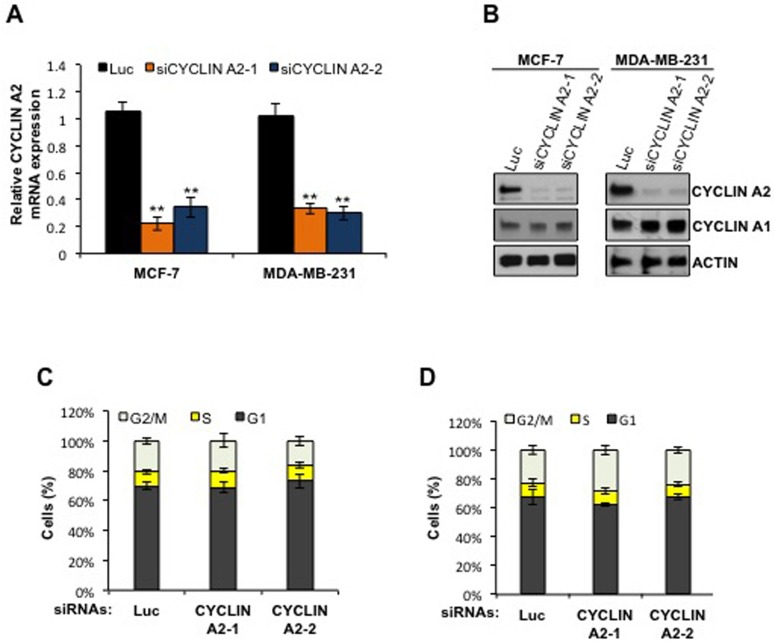
Cyclin A2 mRNA and protein abundance in cyclin A2 siRNA-transfected MCF-7 and MDA-MB-231 cells The MCF-7 and MDA-MB-231 cells were transfected with control luciferase (Luc) siRNA or two independent siRNAs targeting cyclin A2 (siCYCLIN A2-1 and siCYCLINA2-2) and the mRNA **A.,** and protein **B.** abundance of cyclin A2 was examined by qRT-PCR and Western blotting, respectively. Cell cycle profile of Luc- and cyclin A2 siRNA-transfected MCF-7 and MDA-MB-231 cells is shown in **C.** and **D.**, respectively.

To determine the cellular roles of cyclin A2 in breast cancer cells, cyclin A2 was depleted in human MCF-7 and MDA-MB-231 breast cancer cell lines using siRNAs that target cyclin A2. Cyclin A2 mRNA and protein expression was markedly reduced in the cyclin A2 siRNA-transfected cells compared to the non-silencing siRNA (luciferase siRNA)-transfected cells (Figure 1A). However, the expression of cyclin A1, an alternative A-type cyclin, was unaffected by the transfection of cyclin A2 siRNAs, indicating the specificity of the siRNAs in targeting cyclin A2 in the cells (Figure 1B). Cyclin A2 regulates G1/S and G2/M transition, however, depletion of cyclin A2 in both MCF-7 and MDA-MB-231 cells did not affect the cell cycle profile of the cells (Figure 1C and 1D).

### Cyclin A2 depletion results in reduced homologous recombination and increased DNA damage

**Figure 2:**
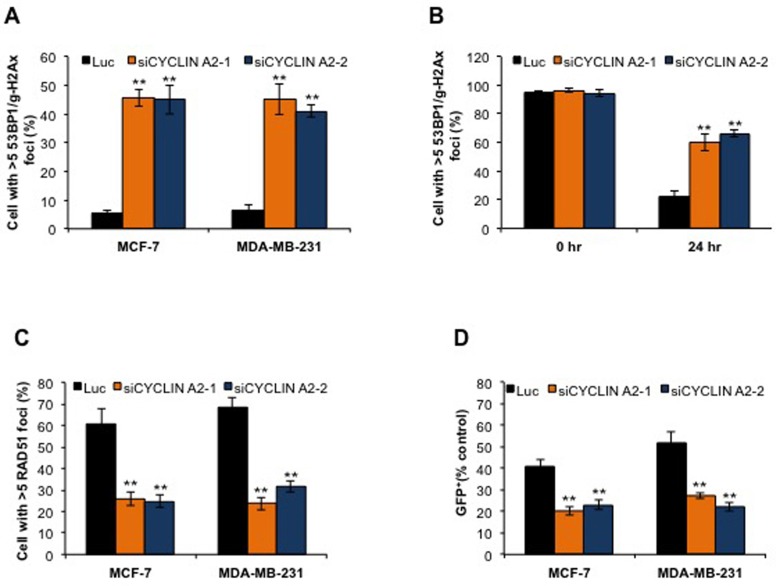
Cyclin A2 depletion induces DNA double-strand breaks and defects in HR repair **A.** After 48 hr siRNA transfection, MCF-7 and MDA-MB-231 cells were immunostained for γ-H2Ax and 53BP1, and cells with >5 γ-H2Ax/53BP1 co-localized foci were counted to measure the incidence of DSBs. **B.** Control (luciferase) siRNA- or cyclin A2 siRNA-transfected MCF-7 cells were exposed to ionizing irradiation (1 Gy) and the efficiency of DNA DSB repair was examined by counting cells with >5 γ-H2Ax/53BP1 co-localized foci after 24 hr. **C.** The cells were transfected with control luciferase (Luc), or cyclin A2 siRNAs. 48 hr after transfection, the cells were irradiated with 10 Gy of ionizing radiation and stained to detect RAD51 foci. **D.** MCF-7-DR-GFP cells transfected with pCβASceI plasmid plus control luciferase (Luc), or cyclin A2 siRNAs were analyzed by flow cytometry for GFP fluorescence after 72 h transfection. Data in (D) were normalized to luciferase-transfected cells for each experiment. Mean ± SEM., *n* = 3; **P < 0.001, Unpaired *t* test.

We monitored the incidence of DNA DSBs to determine whether or not cyclin A2 deficiency induces DNA damage in the cells. This was done by co-immunostaining the cells with γH2Ax and 53BP1 antibodies. The rate of DSBs in the luciferase siRNA-transfected cells was only 5%; while that of cyclin A2 siRNA-transfected cells was 46%, suggesting that cyclin A2 has a major role in safeguarding the cells from DNA damage (Figure 2A). To examine if the increased DSBs was due to decreased repair of the breaks, we induced DNA damage in both luciferase siRNA- and cyclin A2 siRNA-transfected cells using ionizing irradiation (1 Gy) and examined the rate of DNA repair at various time points. About 80% of the luciferase siRNA transfected cells repaired breaks by 24 hr, whereas this was only 36% for cyclin A2-depleted cells at 24 hr (Figure 2B). This suggests that the increased occurrence of DSBs in the cyclin A2 depleted cells was due to reduced repair of the breaks. To determine whether cyclin A2 knockdown affected HR, we evaluated the assembly of RAD51 foci at double-strand breaks sites that were induced by ionizing radiation (1 Gy). We also evaluated the ability of cells to perform HR repair of a genomically integrated DR-GFP substrate (composed of tandem inactive green fluorescent protein (GFP) fragments that flank an I-SceI restriction enzyme site). Depletion of cyclin A2 was found to inhibit ionizing radiation-induced RAD51 foci formation, and the HR repair of DR-GFP substrate, following I-SceI expression (Figure 2C and 2D). Collectively, these results show that cyclin A2 insufficiency disrupts HR repair in breast cancer cells.

### Cyclin A2 deficiency causes reduced abundance of MRE11 protein

**Figure 3:**
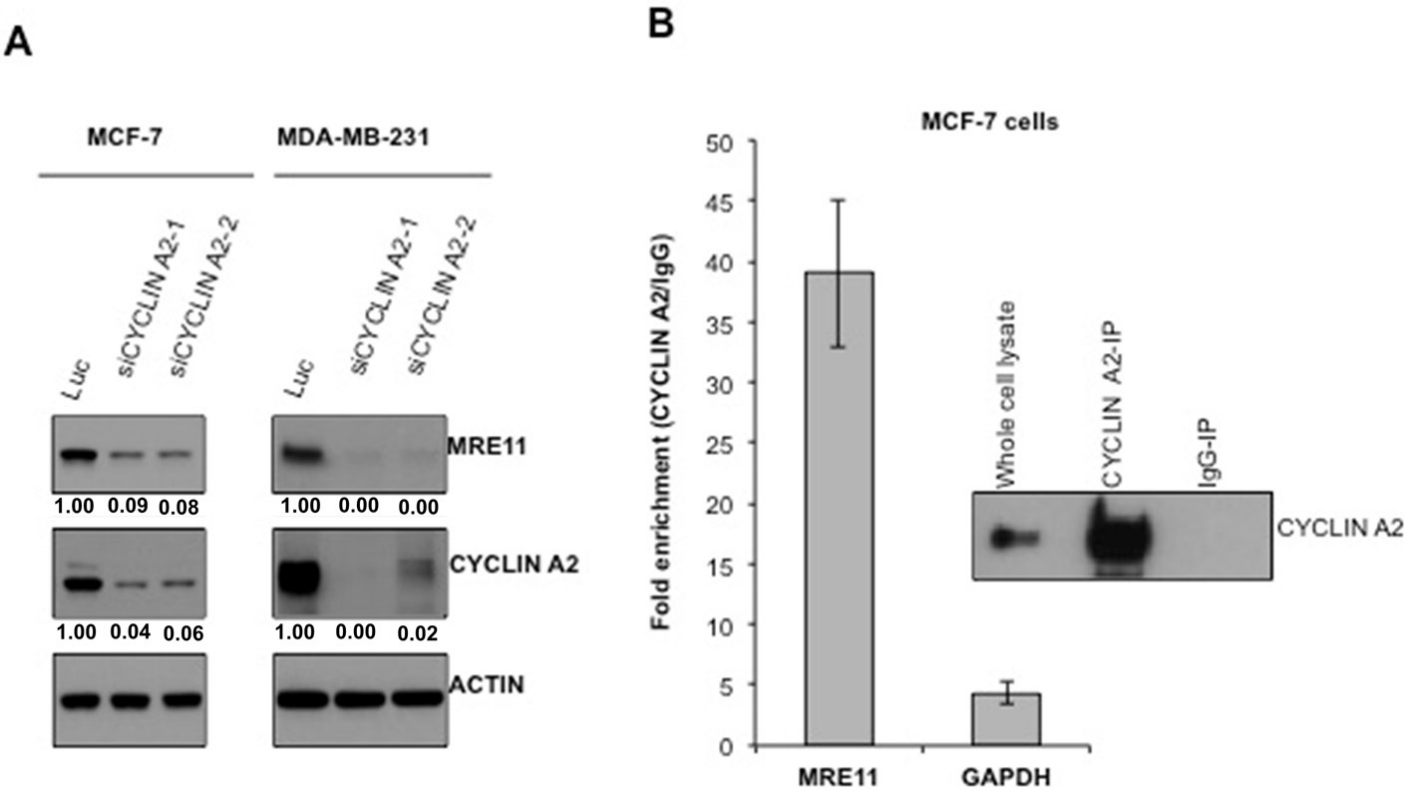
Cyclin A2 interacts with MRE11 mRNA and loss of cyclin A2 reduces MRE11 abundance **A.** After 48 hr of siRNA transfection, the cells were lysed and immunoblotted with indicated antibodies. Actin was used as a loading control. **B.** Cyclin A2 was immunoprecipitated from untransfected MCF-7 cells and co-precipitated MRE11 mRNA was quantified by qRT-PCR.

Cyclin A2 regulates abundance of MRE11 protein through an interaction with MRE11 mRNA in mouse embryonic fibroblast and human fibroblast cells [9]. To determine whether the observed reduction of DNA repair and HR defect, upon cyclin A2 knockdown, was a result of reduced MRE11 levels, MRE11 protein abundance was checked by immune-blotting. As shown in Figure 3A, loss of cyclin A2 markedly decreased MRE11 abundance in both MCF-7 and MDA-MB-231 cells. Consistent with the previous study [9], cyclin A2 interacted with MRE11 mRNA in MCF-7 cells Figure 3B. Together, these results suggest that cyclin A2 is a positive regulator of MRE11 in breast cancer cells.

### Ectopic expression of MRE11 partially corrected HR defects in cyclin A2 depleted cells

**Figure 4:**
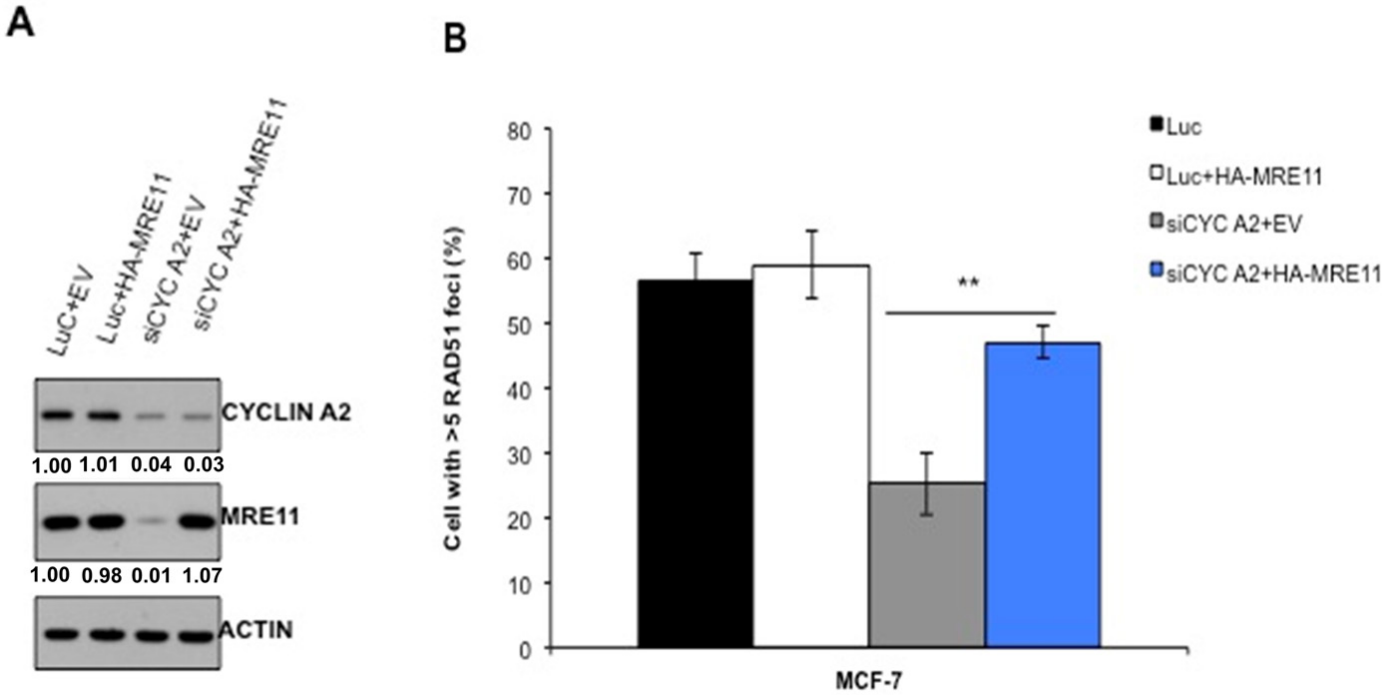
Restoration of MRE11 expression in cyclin A2 depleted cells corrects defect in HR repair The MCF-7 cells were transfected with control luciferase siRNA plus empty vector (EV) or HA-MRE11 expression vector or cyclin A2 siRNA plus empty vector (EV) or HA-MRE11 expression vector. After 48 h transfection, portion of the cells were lysed and immunoblotted with indicated antibodies **A.** Actin was used as a loading control. The remaining cells were reseeded and irradiated with 10 Gy of ionizing radiation and stained to detect RAD51 foci **B.** Mean ± SEM, *n* = 3; *P < 0.001*,* Unpaired *t* test.

To define if loss of MRE11 is the determinant of HR defect in cyclin A2-depleted cells, we ectopically expressed MRE11 in cyclin A2-depleted cells and examined the HR repair of the DR-GFP substrate following I-SceI expression. MRE11 expression in cyclin A2-depleted MCF-7 cells partially rescued the HR defects (Figure 4), suggesting that cyclin A2 regulates HR repair through promoting MRE11 expression.

### Cyclin A2 insufficiency perturbs RAD51 protein stability and causes reduced abundance of RAD51 protein

**Figure 5:**
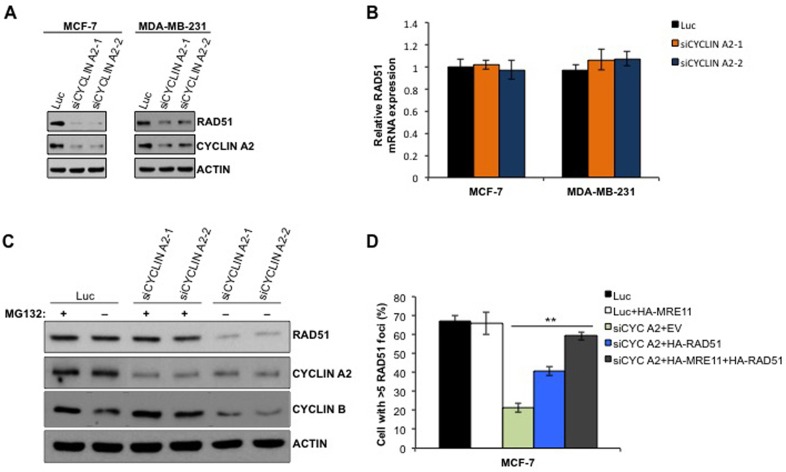
Cyclin A2 depletion causes decrease in RAD51 abundance **A.** Western blots of indicated proteins in cyclin A2 depleted MCF-7 and MDA-MB-231 cells. **B.** qRT-PCR analysis of RAD51 mRNA expression. **C.** Western blot analysis of indicated proteins in MG132-untreated and -treated control luciferase siRNA or or cyclin A2 siRNA transfected cells. **D.** The MCF-7 cells were transfected with control luciferase siRNA plus empty vector (EV) or HA-MRE11 or HA-RAD51 or HA-MRE11+HA-RAD51 expression vector or cyclin A2 siRNA plus empty vector (EV) or HA-MRE11 or HA-RAD51 or HA-MRE11+HA-RAD51 expression vector. The cells were irradiated with 10 Gy of ionizing radiation and stained to detect RAD51 foci.

To determine if the reduced RAD51 foci formation in the cyclin A2 depleted cells was due to decreased abundance of RAD51 protein (Figure 2C), we quantified the RAD51 levels in cyclin A2 siRNA-transfected MCF-7 and MDA-MB-231 cells. Cyclin A2 depletion caused reduction in RAD51 levels in both the cell types (Figure 5A). The reduced RAD51 levels was not due to decrease in RAD51 mRNA levels (Figure 5B), suggesting that cyclin A2 regulation of RAD51 is not at the transcriptional level. To assess if proteasome degradation of RAD51 was responsible for its reduction by cyclin A2 depletion, we treated control and cyclin A2 siRNA-transfected cells with proteosome inhibitor MG132. Treatment with MG132 restored RAD51 protein abundance in cyclin A2 depleted cells (Figure 5C). Together, these results indicate that cyclin A2 functions to prevent proteosomal degradation of RAD51 to maintain sufficient levels of the protein to carry out HR.

### Ectopic expression of RAD51 rescued HR defects in cyclin A2 depleted cells

To determine if loss of RAD51 contributes to the HR defect in cyclin A2-depleted cells, we ectopically expressed RAD51 in cyclin A2-depleted cells and examined the HR repair of the DR-GFP substrate following I-SceI expression. RAD51 expression in cyclin A2-depleted MCF-7 cells partially rescued the HR defects (Figure 5D). While, co-expression of RAD51 and MRE11 fully corrected the defect (Figure 5D), suggesting that cyclin A2 regulates HR repair through promoting MRE11 and RAD51 expression.

### Cyclin A2 depletion sensitizes breast cancer cells to DNA damaging agent and PARP inhibitors

**Figure 6:**
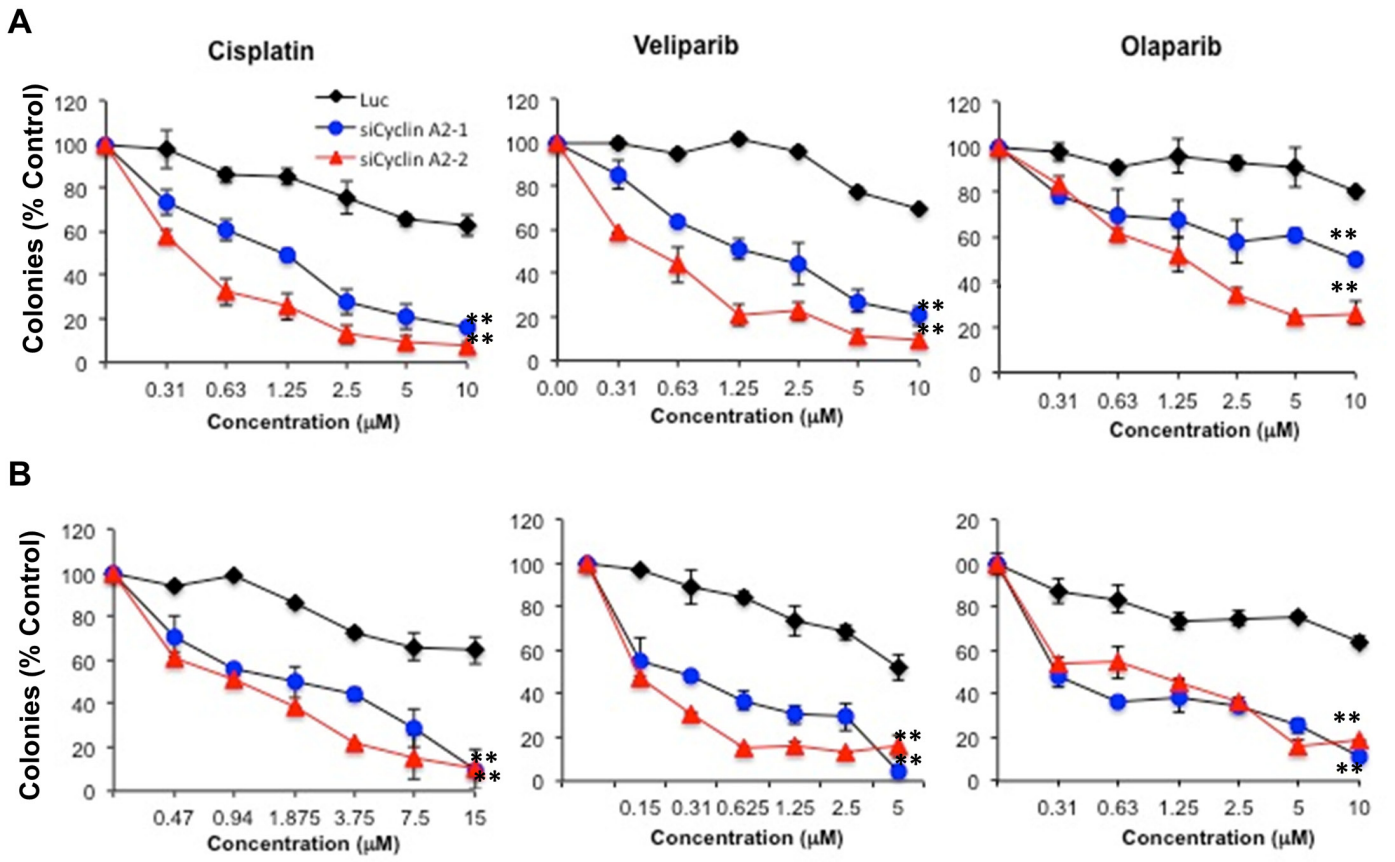
Sensitivity of cyclin A2 depleted MCF-7 and MDA-MB-231 cells to DNA damaging agent and PARP inhibitors The MCF-7 cells **A.** and MDA-MB-231 cells **B.** were transfected with control luciferase or two independent siRNAs for cyclin A2. After 48 hr posttransfection, the cells were used in clonogenic assays. For clonogenic assays, cells were plated (600 cells per well) and allowed to adhere for 4-6 h, treated with cisplatin for 24 h, washed, and re-fed. Alternatively, cells were treated with veliparib or olaparib continuously and allowed to form colonies for 9-14 days. Colonies (>50 cells) were stained with Coomassie Blue and manually counted. Results shown are representative of 3 independent experiments.

HR defects sensitize cancer cells to DNA crosslinkers and PARP inhibitors [29]. To determine whether loss of cyclin A2 sufficiently perturbs HR to sensitize breast cancer cells to DNA crosslinkers and PARP inhibition, we treated the cells with DNA cross linker, cisplatin, and two PARP inhibitors, veliparib (ABT-888) and olaparib (AZD2281). Consistent with impairment in RAD51 foci formation and HR defect, cyclin A2 depleted cells were more sensitive to both veliparib and olaparib (Figure 6).

## DISCUSSION

Cells constantly encounter DNA damage and, therefore, efficient DNA repair is critical to maintain genomic stability in cells [30]. Increasing evidence indicates that defects in HR-mediated DNA repair underlie sporadic and hereditary tumorigenesis, and that HR deficient tumors are specifically vulnerable to certain DNA damaging agents [31]. Moreover, HR-defective tumors are hypersensitive to PARP inhibitors through synthetic lethal interactions, a concept that is currently being tested in clinical trials [31-33]. HR deficiency is, therefore, largely considered to be a diagnostic criterion per se if suitable biomarkers become available to identify such tumors.

Cyclin A2, whose expression is frequently altered in diverse human cancers [18-28], was first identified over 30 years ago; however, to-date, not much has been done to potentially exploit it for cancer therapeutics. Our studies to address the consequence of loss of cyclin A2 in breast cancer cells identified that cyclin A2 deficiency causes high rates of DSBs in the cells (Figure 2A). DSBs are the most lethal type of DNA damage. If left unrepaired, DSBs lead to chromosome segregation defect in the form of chromatin bridges and contribute to genomic instability in cells. DSBs occur through multiple mechanisms. Increased DSBs in the cyclin A2-depleted MCF-7 and MDA-MB-231 cells are likely resulted from the more frequent stalling of the replication forks and impairment in the timely restart of the stalled fork [9] and reduced repair of the breaks (Figure 2B)

Screening for underlying defects revealed that the abundance of MRE11 protein was markedly reduced in cyclin A2 depleted cells (Figure 3A). MRE11 is a key component of the MRN complex that consists of two other proteins, RAD50 and NBS1 [10]. The MRN complex acts upstream of ATM and ATR, and is the major sensor of the DSBs [10, 15]. In addition to having a role in the restart of stalled replication fork, the complex is important for DNA end resection, which is one of the earliest steps in the HR-mediated DNA repair [10, 34]. In mouse embryonic fibroblast and human fibroblast cells, cyclin A2 has been shown to enhance MRE11 abundance through a CDK-independent RNA binding property [9]. Once bound to the MRE11 mRNA, cyclin A2 favors its translation through an interaction with eukaryotic translation initiation factor, eIF4A2 [9]. We were able to find a strong interaction between cyclin A2 and MRE11 mRNA in the control siRNA-transfected MCF-7 cells (Figure 3B), suggesting that the RNA binding property of cyclin A2 is also conserved in breast cancer cells and that the decreased abundance of MRE11 protein in the cyclin A2 siRNA-transfected cells may be due to the lack of cyclin A2 binding onto the MRE11 mRNA.

Our further analyses of cyclin A2 exposed that depleting cyclin A2 reduced RAD51 foci formation and HR repair, two phenotypes that are associated with HR defects (Figure 2C and D). Reduced RAD51 foci formation in cyclin A2 deficient cells was associated with low levels of RAD51 protein, resulting from its increased proteasome degradation (Figure 5). The evidence that MRE11 and RAD51 overexpression corrected HR defects in MCF-7 cells suggests that cyclin A2 regulation of HR repair is mediated through MRE11 and RAD51 axis (Figure 5). Consistent with these findings, depleting cyclin A2 sensitized MCF-7 and MDA-MB-231 cells to DNA damaging agent and PARP inhibitors (Figure 6), a drug class that has shown success against breast cancer.

Taken together, our present study identifies cyclin A2 as a novel determinant of HR efficiency in human breast cancer cells. We demonstrate that cyclin A2 plays a vital role in maintaining adequate levels of MRE11 and RAD51 proteins for proper functioning of HR repair and that its loss sensitizes the breast cancer cells to DNA damaging agents and PARP inhibitors. These results provide important basis for developing cyclin A2 as a biomarker for predicting response to therapy in breast cancer patients.

## MATERIALS AND METHODS

### Antibodies, drugs, and siRNAs

Antibodies and suppliers were as follows: mouse anti-cyclin A2 (1:5,000, E23.1, Abcam); mouse anti-actin (1:5,000, A5441/clone AC-15, Sigma-Aldrich); mouse anti-MRE11 (1:100, SC-135992, SantaCruz); rabbit anti-RAD51 (Calbiochem, PC130), horseradish peroxidase-conjugated anti-mouse or anti-rabbit immunoglobulin G (Cell Signaling Technology). Veliparib (ABT-888) and olaparib (AZD2281) were purchased from Selleck Chemicals. siRNAs designed to target human cyclin A2 was purchased from Dharmacon. An siRNA targeting luciferase that has no specificity to any human genes was used as the negative transfection control throughout the experiments.

### Cell lines, cell culture, and transfections

MCF-7 and MDA-MB-231 cells were obtained from the American Type Culture Collection (ATCC, Rockville, MD). The cells were maintained in cell culture media and conditions recommended by the American Type Culture Collection. MCF-7 cells were grown in DMEM medium containing 10% (V/V) FBS without antibiotics at 37°C in a humidified atmosphere containing 95% air and 5% CO_2_. MDA-MB-231 cells were grown in L-15 medium containing 10% (V/V) FBS without antibiotics at 37°C.

### Cell cycle analysis

For the cell cycle analysis by propidium iodide, cells were harvested and fixed in 95% ethanol for 5 min on ice. Cells were washed and treated with RNase before being stained with propidium iodide (100 µg/ml in 1% sodium citrate). After 15 min incubation in the dark, cell cycle profiles were analyzed by flow cytometry.

### Quantitative real-time PCR

Total RNA was extracted from the cells using miRNeasy Mini Kit (Qiagen). cDNA synthesis was carried out with SuperScript III reverse transcription (Invitrogen). The quantitative real-time PCR performed using iTaq Universal SYBR Green Supermix (Bio-Rad). The qRT-PCR primers used were as follows: Cyclin A2: Forward: 5’-TTATTGCTGGAGCTGCCTTT-3’, Reverse: 5’-CTCTGGTGGGTTGAGGAGAG-3’; GAPDH: Forward: GAGTCAACGGATTTGGTCGT, Reverse: TTGATTTTGGAGGGATCTCG. The mRNA expression levels of were quantified by measuring the threshold cycle (Ct).

### DR-GFP HR assays

Measurement of the repair of I-*Sce*I-generated DSB, by using DR-GFP system, was described previously [35]. In brief, after stable transfection and genomic integration of DR-GFP plasmid in MCF-7 cells, 50 μg of I-*Sce*I expression vector (pCBASce) or control empty vector was transfected by electroporation, using a BTX ECM 830 square wave electroporator (Holliston, MA). This was followed by transfection of cells with cyclin A2 siRNAs and incubation for 48 hours. For the measurement of homologous recombination events, % green fluorescent protein (GFP)-positive cells were quantitated by flow cytometric analysis of live cells that were trypsinized and recovered. Specifically, homologous recombination events were calculated from plots of FL-1 (GFP) and FL-3 (autofluorescence) on the ordinate and abscissa, respectively, with a gate set to include 0.10% GFP^+^ cells electroporated with an empty vector as background.

### Clonogenic assays

Cells were transfected with cyclin A2 siRNAs and clonogenic assay was performed as described before [36].

### Immunoblotting

Cells were lysed and immunoblotting was performed as described before [36, 37].

### RNA immunoprecipitation

RNA immunoprecipitation and qRT-PCR for MRE11 mRNA was performed as described before[9]. The qRT-PCR primers used were: MRE11: Forward: 5’-AGAGCCGAACTGGACTTGAA-3’, Reverse: 5’-GGTCAGTCAAGCTCCTCTGG-3’.
